# IAM Chromatographic Models of Skin Permeation

**DOI:** 10.3390/molecules27061893

**Published:** 2022-03-15

**Authors:** Anna W. Sobańska, Elżbieta Brzezińska

**Affiliations:** Department of Analytical Chemistry, Faculty of Pharmacy, Medical University of Lodz, ul. Muszyńskiego 1, 90-151 Lodz, Poland; elzbieta.brzezinska@umed.lodz.pl

**Keywords:** Immobilized Artificial Membrane, HPLC, calculated descriptors, skin permeation

## Abstract

Chromatographic retention factor log ***k_IAM_*** obtained from IAM HPLC chromatography with buffered aqueous mobile phases and calculated molecular descriptors (surface area—***S_a_***; molar volume—***V_M_***; polar surface area—***PSA***; count of freely rotable bonds—***FRB***; H-bond acceptor count—***HA***; energy of the highest occupied molecular orbital—***E_HOMO_***; energy of the lowest unoccupied orbital—***E_LUMO_***; and polarizability—***α***) obtained for a group of 160 structurally unrelated compounds were tested in order to generate useful models of solutes’ skin permeability coefficient log ***K_p_***. It was established that log ***k_IAM_*** obtained in the conditions described in this study is not sufficient as a sole predictor of the skin permeability coefficient. Simple put, potentially useful models based on log ***k_IAM_*** and readily available calculated descriptors, accounting for 85 to 91% of the total variability, were generated using Multiple Linear Regression (MLR).The models proposed in the study were tested on a group of 20 compounds with known experimental log ***K_p_*** values.

## 1. Introduction

Transepidermal absorption is an important route of chemicals’ entry into a human body. The skin permeability coefficient ***K_p_*** is defined according to Equation (1):

(1) $$K_{p}=\frac{K_{m}D}{h}$$

where ***K_m_*** is the partition coefficient between the stratum corneum and the vehicle; ***D*** is the effective compound’s diffusion coefficient through the stratum corneum; and ***h*** is the diffusional pathlength.

The experimental values of skin permeability coefficients obtained in vivo (on human volunteers), ex vivo (on excised human skin), or even on animal models [1] are scarce and often inconsistent due to variations in properties of different skin specimen; there are also some ethical considerations related to such models. For these reasons, several in vitro or in silico skin permeation models have been developed [2]. One of the most frequently cited in silico skin permeability models, based on just two descriptors known to have a very strong influence on compounds’ ability to cross biological barriers, namely lipophilicity (expressed as octanol-water partition coefficient ***K_ow_***) and molecular weight (***M_w_***), has been proposed by Potts (Equation (2)) [3]:

log ***K_p_*** = −2.80 + 0.66 log ***K_ow_*** − 0.0056 ***M_w_*** (2)

The Potts model is widely acknowledged due to its simplicity [4], although it is criticized by some research studies because it gives erroneous results for compounds of extreme properties (very hydrophilic or lipophilic; non-hydrogen bonding or very strongly hydrogen-bonding) [5,6,7,8]. However, the predictions made using Pott’s model are sufficiently good for the majority of drug-like compounds [9,10], thus this model is a popular tool in drugs’ ADMET studies and calculations based on it are offered by some popular ADMET prediction software packages [11,12]. Other proposed theoretical ***K_p_*** models are based on the descriptors, such as the melting point, McGowan’s characteristic volume, Abraham’s solvation parameters or H-bonding properties (total H-bond count, H-bond acceptor count, and H-bond donor count), and total nitrogen and oxygen atom count [13,14,15,16,17,18,19,20,21,22,23]. QSAR studies of skin permeation published to date prove that transdermal absorption is a complex property and there are several factors contributing to it [4,7,24,25,26,27,28,29]. 

Previous studies demonstrated the usefulness of chromatographic descriptors in skin permeability studies. Liquid chromatographic models of skin permeability are based on the notion that the solutes’ partition between a stationary phase and a mobile phase resembles the partition between the skin and the vehicle.The separation techniques capable of providing chromatographic skin permeability predictors are liquid chromatography (HPLC or TLC), biopartitioning micellar chromatography, micellar electrokinetic chromatography, liposome electrokinetic chromatography, and two-dimensional gas chromatography (GCxGC) [8,30,31,32,33,34,35,36,37,38,39,40]. Chromatographic techniques of skin permeability studies are growing in popularity because of their high throughput, low cost, and good repeatability/reproducibility (the majority of such studies are conducted on commercially available stationary phases). 

In our earlier studies, we proposed models of ***K_p_*** based on calculated molecular parameters and RP-18 TLC-derived descriptors (***R_M_*** or ***R_M_***/***V_M_***) [23,41]:

log ***K_p_*** = −1.66 (±0.24) − 0.011 (±0.005) ***PSA*** + 0.24 (±0.05) ***HD*** − 0.0036 (±0.0017) ***V_M_*** + 2.01 (±0.24) ***R_M_*** (steroids, *n* = 16, R^2^ = 0.99, R^2^_adj._ = 0.98, F = 229.0, *p* < 0.01, s_e_ = 0.18) (3)

log ***K_p_*** = −1.65 (±0.90) − 0.37 (±0.03) (***N+O***) + 0.13 (±0.03) log ***D*** + 90.4 (±43.0) (***R_M_***/***V_M_***) − 0.0000058 (±0.0000019) ***E_T_*** + 0.029 (±0.01) ***E_h_*** (structurally diverse compounds, *n* = 60, R^2^ = 0.87, R^2^_adj._ = 0.85, F = 70.9, *p* < 0.01, s_e_ = 0.40) (4)

where ***R_M_*** = log (1/***R_f_*** − 1) [42] and ***R_f_*** values were collected on the RP-18 stationary phase with acetonitrile/pH 7.4 phosphate-buffered saline 70:30 (*v*/*v*) as a mobile phase; log ***D*** is the distribution coefficient; ***PSA*** is the polar surface area (Å^2^); ***HD*** is the H-bond donors count; ***V_M_*** is the molar volume (Å^3^); ***E_T_*** is the total energy (kcal/mol); ***E_h_*** is the hydration energy (kcal/mol); and (***N + O***) is the total oxygen and nitrogen atom count.

We have also studied the skin permeability of organic sunscreens using RP-18 TLC-derived descriptors obtained with mobile phases containing different organic modifiers [43].

Thanks to the ability of stationary phases based on phosphatidylcholine covalently linked to aminopropyl silica to mimic the natural membrane bilayer (or, to be precise, a half of it), Immobilized Artificial Membrane (IAM) chromatography performed on such sorbents has been used to predict physico-chemical and biological properties of solutes for many years [44]. The relationships between the IAM chromatographic retention factor (***k_IAM_***) and the skin permeability coefficient have been studied for small groups of compounds (*n* = 10 to 32) and the resulting dependencies are mostly univariate (linear or quadratic) [30,31,34,36], the exceptions being the study in which McGowan’s characteristic volume ***V*** was incorporated in a model alongside with log ***k_IAM_*** [31]:

log ***K_p_*** = −3.58 + 2.56 log ***k_IAM_*** − 1.12 ***V*** (*n* = 32, R^2^ = 0.74) (5)

Additionally, the model proposed by Barbato, based on a combination of an IAM chromatographic descriptor and the octanol-water partition coefficient log ***K_ow_***, is as follows [30]:

log ***K_p_***= −2.136 Δlog***k_w_^IAM^***+ 0.037 log ***K_ow_***− 2.373 (*n* = 10, R^2^ = 0.94) (6)

where Δlog***k_w_^IAM^*** is the difference between log ***k_w_^IAM^*** measured and predicted on the basis of log ***K_ow_***.

In this study it was our intention to investigate the potential of immobilized artificial membrane (IAM) chromatography in skin permeability studies of a large group of solutes from different chemical families.We hoped to provide simple and practical models based on the IAM chromatographic retention factors and calculated physico-chemical descriptors that could be used to predict the skin permeability coefficient of solutes by researchers both in drug discovery and environmental toxicology fields.

## 2. Results and Discussion

The experimentally determined values of ***K_p_*** were available for only some drugs within the studied group.For this reason, the models of skin permeability involving IAM chromatographic and calculated descriptors were generated and validated using ***K_p_*** values obtained in silico with the EpiSuite software (DERMWIN v. 2 module; log ***K_p_***^EPI^), which is recommended by the US Environmental Protection Agency [12,45] and was tested on a sub-group of 20 solutes whose experimental log ***K_p_*** values are known (log ***K_p_***^exp^) [29]. The estimation methodology used by DERMWIN was based on the above-mentioned Equation (2) [3]. The values of log ***K_p_***^EPI^ obtained using DERMWIN are given in Table 1.

The compounds ***1*** to ***160*** were chromatographed on the IAM stationary phase using buffered aqueous mobile phases as described in Section 3. The retention factors (log ***k_IAM_***) were compiled from the published literature by Sprunger et al. [46] whose main objective was to propose an IAM chromatographic retention model based on Abraham’s solvation parameters. In our investigations, we focused mainly on drugs (that are or potentially could be administered transdermally) and environmentally relevant compounds (organic pollutants whose skin absorption can be a possible route of exposure). Log ***k_IAM_*** values taken from Reference [46] were correlated with the log ***K_p_***^EPI^ values presented in Table 1. Unfortunately, the resulting linear correlation (7) is poor, with R^2^ = 0.46.

log ***K_p_***^EPI^ = −2.98 (±0.07) + 0.52 (±0.04) log ***k_IAM_*** (*n* = 160, R^2^ = 0.46, R^2^_adj._ = 0.45, MSE = 0.32, F = 132.7, *p* < 0.01, s_e_ = 0.57) (7)

Previous studies of the relationships between log ***K_p_*** and log ***k_IAM_*** (e.g., Equation (8)) for small groups of compounds [31,34,36] failed to provide general chromatographic models applicable to molecules from different chemical classes:

log ***K_p_***= −5.154 + 1.443 log ***k^IAM^*** (*n* = 32, R^2^ = 0.26) (8)

From this (and Equation (5) [31]), it was concluded that log ***k_IAM_*** obtained as described in Section 3 is not sufficient as a sole predictor of log ***K_p_***. At this point, it was decided to seek a multivariate linear relationship that would meet the following requirements: (i) give the best possible fit with the log ***K_p_***^EPI^ reference values; (ii) fit the experimental log ***K_p_***^exp^ values for a subgroup of compounds whose experimental skin permeability data are available (preferably from a single source to avoid possible discrepancies between experimental data collected by different protocols); and (iii) be as simple as possible and contain the minimum number of independent variables needed to generate models of reasonable predictive power without the risk of overfitting.These goals were achieved in our study by taking the following steps:

- Generating a well-fitting model based on a relatively large number of independent variables selected by forward stepwise multiple regression;
- Validation of the model using two randomly selected subsets of compounds, namely a training set (*n* = 120) and a test set (*n* = 40);
- Validation of the initial model using experimental log ***K_p_***^exp^ data for a subset of compounds (*n* = 20);
- Analysis of every step of multiple stepwise regression in order to eliminate redundant independent variables; and
- Building a new model based on a reduced set of independent variables and its validation as described above.

**Figure 1:**
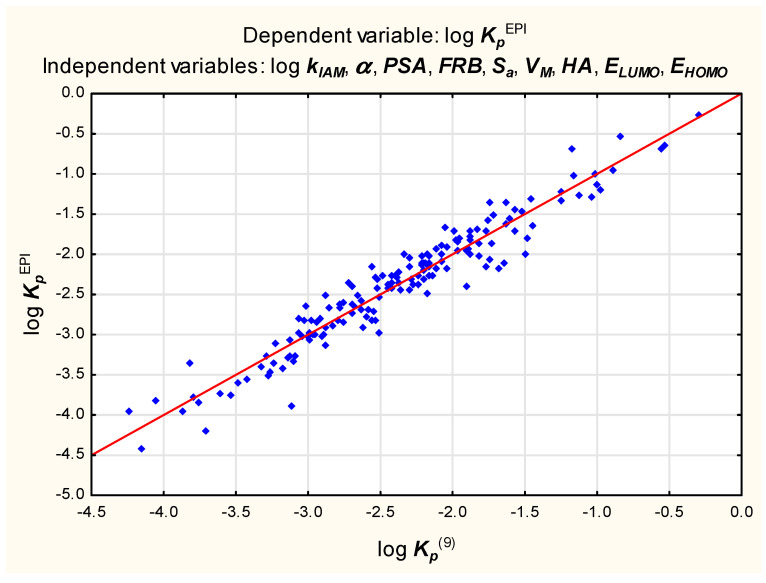
Equation (9)—reference vs. predicted log ***K_p_*** values.

In the first step of the multiple regression analysis, the calculated physicochemical parameters presented in Table 2 were incorporated by forward stepwise multiple regression (Equation (9), Figure 1):

log ***K_p_***^EPI^ = −1.66 (±0.30) + 0.75 (±0.03) log ***k_IAM_*** − 0.0034 (±0.002) ***PSA*** + 0.033 (±0.016) ***FRB*** − 0.089 (±0.035) ***HA*** + 0.0090 (±0.003) ***V_M_*** − 0.092 (±0.021) ***α*** + 0.035 (±0.028) ***E_HOMO_***− 0.031 (±0.024) ***E_LUMO_*** − 0.0022 (±0.0008) ***S_a_*** (*n* = 160, R^2^ = 0.96, R^2^_adj._ = 0.92, MSE = 0.043, F = 216.6, *p* < 0.01, s_e_ = 0.21) (9)

The model (9) was validated using the holdout method in which data points are assigned to two sets usually called the training set and the test set. The size of each set is arbitrary (the test set is usually smaller than the training set). The group of 160 studied compounds was divided into two subsets: a training set (***1*** to ***120***) and a test set (***121*** to ***160***).The Equation (10) generated for the training set and containing the same independent variables as Equation (9) is as follows:

log ***K_p_***^EPI^ = −1.71 (±0.35) + 0.76 (±0.03) log ***k_IAM_*** − 0.0042 (±0.002) ***PSA*** + 0.038 (±0.02) ***FRB*** − 0.074 (±0.035) ***HA*** + 0.0087 (±0.003) ***V_M_*** − 0.11 (±0.02) ***α*** + 0.050 (±0.029) ***E_HOMO_*** − 0.028 (±0.023) ***E_LUMO_*** − 0.00053 (±0.00138) ***S_a_*** (*n* = 120, R^2^ = 0.95, R^2^_adj._ = 0.94, MSE = 0.032, F = 210.5, *p* < 0.01, s_e_ = 0.18) (10)

The values of log ***K_p_***^(10)^ were calculated for the test set according to Equation (10) and plotted against the reference log ***K_p_***^EPI^ values to furnish a linear relationship (R^2^ = 0.87). The model (9) was also tested on the subgroup of 20 compounds whose log ***K_p_***^exp^ values were available (16 compounds belonging to the training set and four compounds belonging to the test set).The resulting relationship between log ***K_p_***^(9)^ and log ***K_p_***^exp^ is linear, with R^2^ = 0.90.

**Figure 2:**
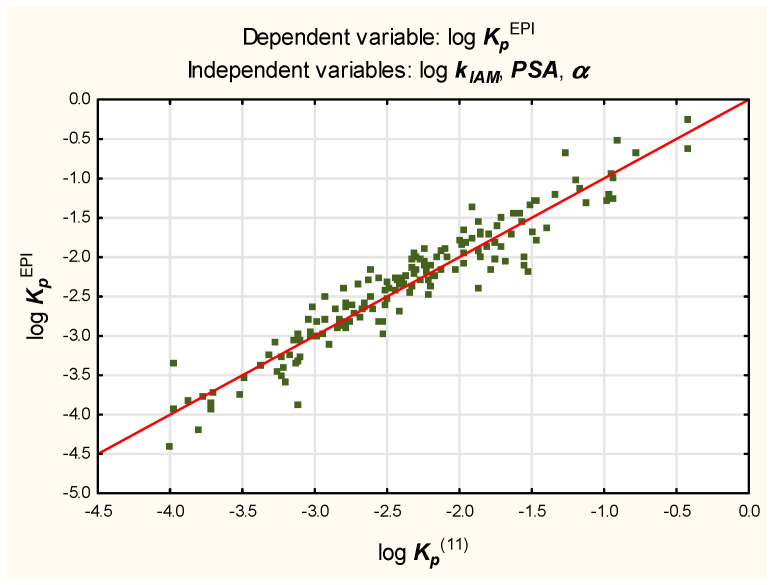
Equation (11)—reference vs. predicted log ***K_p_*** values.

**Figure 3:**
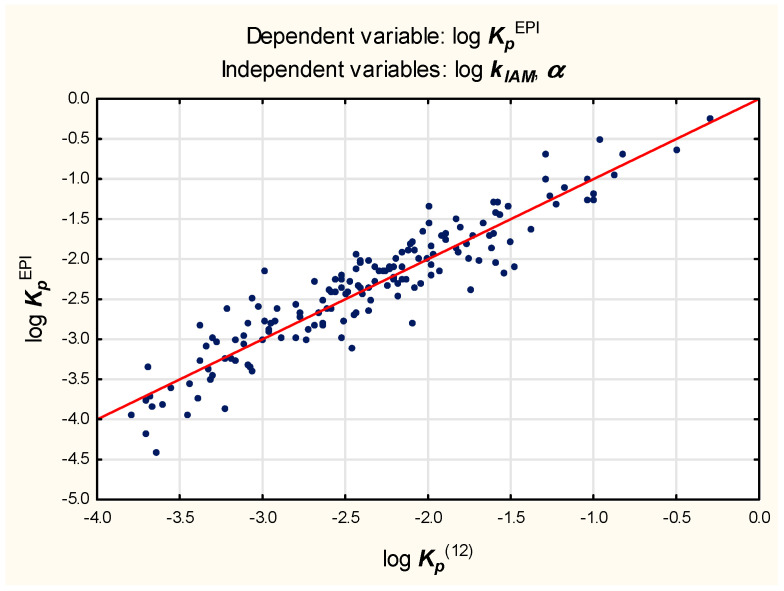
Equation (12)—reference vs. predicted log ***K_p_*** values.

**Figure 4:**
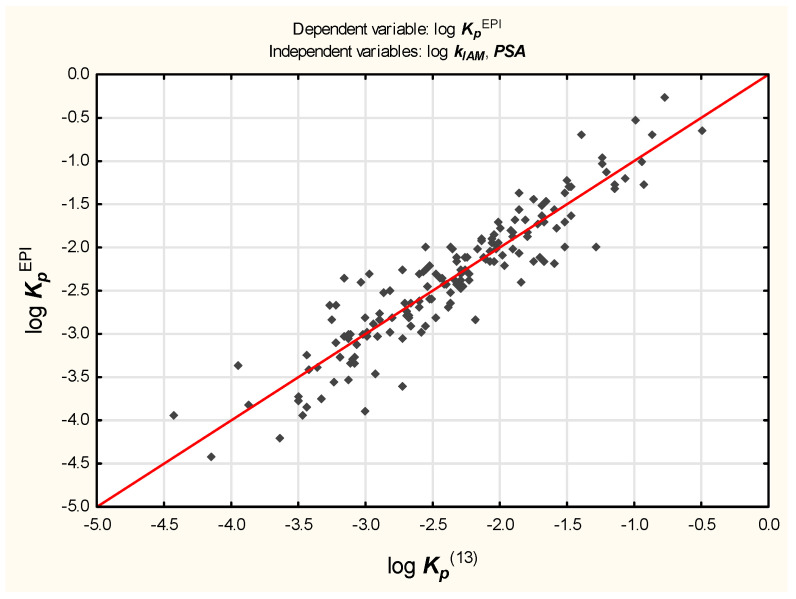
Equation (13)—reference vs. predicted log ***K_p_*** values.

Equation (9), despite encouraging results of validation, was found unsatisfying because it seems over-parameterized; it contains nine independent variables whose contributions, apart from log ***k_IAM_***, ***α*** and ***PSA***, are negligible (log ***k_IAM_***, ***α*** and ***PSA*** account for over 91% of the total variability and the remaining six variables for less than 4%).With so many independent variables, it is also difficult to avoid colinearity.A decision was made to simplify Equation (9) as much as possible; apart from log ***k_IAM_***, only two variables—***PSA*** and ***α***—seem to have a sufficient influence on log ***K_p_*** to justify incorporating them in Equations (11)–(13) (Figure 2, Figure 3 and Figure 4).

log ***K_p_***^EPI^ = −2.22 (±0.04) + 0.75 (±0.03) log ***k_IAM_*** − 0.042 (±0.004) ***α*** − 0.0096 (±0.0011) ***PSA*** (*n* = 160, R^2^ = 0.91, R^2^_adj._ = 0.91, MSE = 0.052, F = 538.9, *p* < 0.01, s_e_ = 0.23) (11)

log***K_p_***^EPI^ = −2.24 (±0.05) + 0.91 (±0.03) log***k_IAM_*** − 0.070 (±0.003) ***α*** (*n* = 160, R^2^ = 0.87, R^2^_adj._ = 0.87, MSE = 0.078, F = 524.7, *p* < 0.01, s_e_ = 0.28) (12)

log ***K_p_***^EPI^ = −2.39 (±0.05) + 0.51 (±0.02) log ***k_IAM_*** − 0.019 (±0.001) ***PSA*** (*n* = 160, R^2^ = 0.85, R^2^_adj._ = 0.85, MSE = 0.088, F = 452.8, *p* < 0.01, s_e_ = 0.30) (13)

The group of 160 studied compounds was divided into two subsets: a training set (***1*** to ***120***) and a test set (***121*** to ***160***). The Equations (14)–(16), generated for the training set and containing the same independent variables as Equations (11)–(13), are as follows:

log ***K_p_***^EPI^ = −2.17 (±0.05) + 0.79 (±0.03) log ***k_IAM_*** − 0.0086 (±0.0012) ***PSA*** − 0.051 (±0.005) ***α*** (*n* = 120, R^2^ = 0.93, R^2^_adj._ = 0.93, MSE = 0.042, F = 490.7, *p* < 0.01, s_e_ = 0.21) (14)

log ***K_p_***^EPI^ = −2.17 (±0.05) + 0.95 (±0.03) log ***k_IAM_*** − 0.077 (±0.004) ***α*** (*n* = 120, R^2^ = 0.90, R^2^_adj._ = 0.89, MSE = 0.060, F = 500.1, *p* < 0.01, s_e_ = 0.25) (15)

log ***K_p_***^EPI^ = −2.41 (±0.05) + 0.53 (±0.03) log ***k_IAM_*** − 0.017 (±0.001) ***PSA*** (*n* = 120, R^2^ = 0.87, R^2^_adj._ = 0.86, MSE = 0.077, F = 375.2, *p* < 0.01, s_e_ = 0.28) (16)

The values of log ***K_p_***^(14)^, log ***K_p_***^(15)^, and log ***K_p_***^(16)^ were calculated for the test set according to Equations (14)–(16) and plotted against the reference log ***K_p_***^EPI^ values to furnish linear relationships (R^2^ = 0.86, 0.79, and 0.83, respectively).The models (11), (12), and (13) were also tested on the subgroup of 20 compounds whose log ***K_p_***^exp^ values were available.The resulting relationships between log ***K_p_***^(11)^, log ***K_p_***^(12)^, and log ***K_p_***^(13)^—as well as log ***K_p_***^exp^—are linear, with R^2^ = 0.88, 0.84, and 0.80, respectively.

Equation (13) involves log ***k_IAM_***, which encodes some important properties responsible for drugs’ absorption (lipophilicity and molecular size), but accounts for only 46% of the total log ***K_p_*** variability and ***PSA***, which is known to influence other absorption phenomena (e.g., transport through the blood–brain barrier and uptake from a gastrointestinal tract) [47,48,49]. A coefficient for ***PSA*** in Equation (13) is negative, which suggests (as already reported, e.g., for the blood–brain barrier passage or oral absorption) that compounds with large polar surface areas are not easily absorbed through skin.However, from the statistical point of view, Equation (11) is superior to Equations (12) and (13), and gives results comparable to those obtained using Equation (9) without the risk of overfitting.

## 3. Materials and Methods

### 3.1. IAM Chromatography

The chromatographic retention factors (log ***k_IAM_***) for the compounds analyzed in this study were compiled by Sprunger et al. [46]. They were obtained on a IAM.PC.DD2 HPLC column using an aqueous mobile phase buffered at pH ≤ 3 for carboxylic acids and in the pH range of 6.5 to 7.5 for other compounds.

### 3.2. Calculated Molecular Descriptors

The molecular descriptors for the compounds investigated during this study were calculated with HyperChem 8.0 utilizing the PM3 semi-empirical method with Polak–Ribiere’s algorithm: total dipole moment—***DM*** (D), surface area (grid)—***S_a_*** (Å^2^), molecular weight—***M_w_*** (g mol^−1^), energy of the highest occupied molecular orbital—***E_HOMO_*** (eV), and energy of the lowest unoccupied molecular orbital—***E_LUMO_*** (eV). Other physicochemical parameters (octanol-water partition coefficient—log ***P***, polar surface area—***PSA*** (Å^2^), H-bond donor count—***HD***, H-bond acceptor count—***HA***, polarizability—***α*** (cm^3^), molar volume—***V_M_*** (cm^3^), and freely rotable bond count—***FRB***) were calculated using ACD/Labs 8.0 software. (***N+O***), which is the total nitrogen and oxygen atom count, was calculated from the molecular structures. The relevant calculated molecular descriptors are given in Table 2. Statistical analysis was done using Statistica v.13 or StatistiXL v.2.

## 4. Conclusions

Multiple regression models for predicting the skin permeability coefficient of structurally diverse compounds were developed.

Due to the limited availability of experimental permeability data for the solutes investigated in this study, the reference skin permeability coefficients were calculated according to a widely accepted theoretical model proposed by Potts. The values of log ***K_p_*** obtained using this model are in good agreement with the experimental data for drug-like compounds.

The newly developed log ***K_p_*** models are based on a set of IAM chromatographic and computational descriptors.The main descriptors, responsible for the variability of log ***K_p_*** in MLR equations, are log ***k_IAM_***, polarizability (***α***), and polar surface area (***PSA***), and the MLR model gains very little in predictive power when other descriptors are incorporated. Linear relationships based on the IAM chromatographic retention factor and readily available calculated physico-chemical parameters give very good results and have the benefit of simplicity. The proposed models may be applied during the early steps of the drug discovery process when different drugs’ physico-chemical and biological properties are often studied in vitro using IAM chromatography and rapid predictions are required. IAM chromatographic and computational studies of the skin permeability of compounds may therefore be of interest to pharmaceutical and medicinal chemists, and to researchers in the area of environmental sciences since many compounds of environmental concern are absorbed through skin.

## Figures and Tables

**Table 1:** IAM HPLC retention factors: reference, experimental, and calculated values of log ***K_p._***

No.	CAS No.		log *k_IAM_*	log *K_p_*^EPI^	log *K_p_*^(9)^	log *K_p_*^(11)^	log *K_p_*^(12)^	log *K_p_*^(13)^	log *K_p_*^exp^
* **1** *	103-84-4	acetanilide	0.48	−2.79	−2.91	−2.83	−2.92	−2.69	
* **2** *	98-86-2	acetophenone	0.76	−2.43	−2.42	−2.43	−2.55	−2.33	
* **3** *	95-55-6	2-aminophenol	0.28	−3.01	−2.97	−3.01	−2.89	−3.11	
* **4** *	100-66-3	anisole	1.06	−2.01	−2.08	−2.07	−2.19	−2.03	
* **5** *	100-52-7	benzaldehyde	0.63	−2.42	−2.52	−2.47	−2.58	−2.39	
* **6** *	55-21-0	benzamide	0.12	−3.06	−3.13	−3.14	−3.10	−3.13	
* **7** *	71-43-2	benzene	0.96	−1.83	−1.98	−1.95	−2.10	−1.91	
* **8** *	100-47-0	benzonitrile	0.77	−2.35	−2.43	−2.41	−2.41	−2.45	
* **9** *	100-51-6	benzyl alcohol	0.35	−2.68	−2.58	−2.67	−2.77	−2.59	
* **10** *	92-52-4	biphenyl	2.88	−1.01	−1.01	−0.93	−1.03	−0.94	
* **11** *	90-11-9	1-bromonaphtalene	2.90	−1.27	−1.12	−0.92	−1.04	−0.93	
* **12** *	591-20-8	3-bromophenol	1.71	−2.03	−1.81	−1.75	−1.68	−1.91	
* **13** *	104-54-1	cinnamyl alcohol	1.02	−2.26	−2.43	−2.39	−2.52	−2.25	
* **14** *	91-64-5	coumarin	0.99	−2.70	−2.55	−2.40	−2.44	−2.38	
* **15** *	108-94-1	cyclohexanone	0.08	−2.82	−2.79	−2.80	−2.94	−2.67	
* **16** *	84-66-2	diethyl phtalate	1.63	−2.44	−2.37	−2.50	−2.39	−2.54	
* **17** *	576-26-1	2,6-dimethylphenol	1.25	−1.92	−2.11	−2.12	−2.15	−2.14	
* **18** *	623-05-2	4-hydroxybenzyl alcohol	0.12	−3.34	−3.10	−3.10	−3.08	−3.08	
* **19** *	95-48-7	2-methylphenol	1.02	−2.12	−2.21	−2.21	−2.23	−2.25	−1.80
* **20** *	106-44-5	4-methylphenol	1.02	−2.12	−2.21	−2.22	−2.23	−2.26	−1.76
* **21** *	91-20-3	naphtalene	2.47	−1.33	−1.25	−1.12	−1.22	−1.15	
* **22** *	90-15-3	1-naphthol	2.08	−1.72	−1.76	−1.64	−1.62	−1.72	
* **23** *	135-19-3	2-naphthol	1.93	−1.82	−1.88	−1.75	−1.76	−1.79	−1.55
* **24** *	88-74-4	2-nitroaniline	1.13	−2.35	−2.72	−2.70	−2.24	−3.16	
* **25** *	100-01-6	4-nitroaniline	1.01	−2.66	−2.78	−2.78	−2.35	−3.22	
* **26** *	98-95-3	nitrobenzene	1.04	−2.27	−2.49	−2.44	−2.20	−2.72	
* **27** *	619-73-8	4-nitrobenzyl alcohol	0.72	−2.83	−3.03	−2.98	−2.67	−3.26	
* **28** *	627-05-4	1-nitrobutane	0.42	−2.41	−2.70	−2.80	−2.59	−3.03	
* **29** *	99-99-0	4-nitrotoluene	1.35	−2.00	−2.34	−2.29	−2.05	−2.56	
* **30** *	108-95-2	phenol	0.68	−2.36	−2.38	−2.38	−2.40	−2.42	−2.09
* **31** *	60-12-8	2-phenylethanol	0.53	−2.59	−2.63	−2.65	−2.79	−2.50	
* **32** *	92-69-3	4-phenylphenol	2.56	−1.63	−1.44	−1.39	−1.37	−1.47	
* **33** *	108-88-3	toluene	1.40	−1.51	−1.71	−1.70	−1.83	−1.69	
* **34** *	106-48-9	4-chlorophenol	1.48	−1.94	−1.89	−1.87	−1.81	−2.02	−1.44
* **35** *	271-89-6	2,3-benzofuran	1.50	−1.69	−1.83	−1.85	−1.89	−1.88	
* **36** *	526-75-0	2,3-dimethylphenol	1.44	−1.84	−1.96	−1.98	−1.98	−2.04	
* **37** *	105-67-9	2,4-dimethylphenol	1.46	−1.96	−1.96	−1.96	−1.96	−2.03	
* **38** *	91-23-6	2-nitroanisole	1.09	−2.51	−2.66	−2.61	−2.34	−2.87	
* **39** *	95-51-2	2-chloroaniline	1.21	−2.26	−2.16	−2.17	−2.12	−2.27	
* **40** *	99-09-2	3-nitroaniline	0.93	−2.67	−2.85	−2.85	−2.42	−3.26	
* **41** *	539-03-7	4-chloroacetanilide	1.27	−2.37	−2.45	−2.32	−2.35	−2.29	
* **42** *	106-47-8	4-chloroaniline	1.16	−2.30	−2.21	−2.21	−2.17	−2.29	
* **43** *	62-53-3	aniline	0.35	−2.73	−2.69	−2.73	−2.77	−2.70	
* **44** *	60-80-0	antipyrine	0.22	−3.61	−3.48	−3.20	−3.55	−2.72	
* **45** *	119-61-9	benzophenone	2.06	−1.71	−1.88	−1.79	−1.92	−1.67	
* **46** *	120-51-4	benzyl benzoate	2.71	−1.36	−1.64	−1.50	−1.51	−1.51	
* **47** *	108-86-1	bromobenzene	1.75	−1.70	−1.57	−1.49	−1.59	−1.51	
* **48** *	68411-44-9	butylbenzene	2.78	−0.52	−0.84	−0.90	−0.96	−0.99	
* **49** *	495-40-9	butyrophenone	1.56	−1.80	−1.95	−1.99	−2.08	−1.92	
* **50** *	58-08-2	caffeine	−0.17	−3.94	−4.24	−3.71	−3.78	−3.47	
* **51** *	108-90-7	chlorobenzene	1.58	−1.55	−1.60	−1.57	−1.67	−1.59	
* **52** *	50-22-6	corticosterone	1.69	−3.46	−3.26	−3.25	−3.30	−2.92	
* **53** *	53-06-5	cortisone	1.29	−3.85	−3.75	−3.72	−3.67	−3.44	
* **54** *	50-28-2	estradiol	2.65	−1.67	−2.05	−1.96	−2.03	−1.80	−2.28
* **55** *	50-27-1	estriol	1.66	−2.80	−3.06	−2.93	−2.98	−2.68	
* **56** *	100-41-4	ethylbenzene	1.81	−1.31	−1.46	−1.48	−1.59	−1.48	
* **57** *	110-00-9	furan	0.32	−2.30	−2.38	−2.42	−2.46	−2.48	
* **58** *	106-24-1	geraniol	1.80	−1.36	−1.74	−1.91	−1.98	−1.86	
* **59** *	1671-75-6	heptanophenone	2.99	−1.13	−1.00	−1.16	−1.18	−1.20	
* **60** *	50-23-7	hydrocortisone	1.30	−3.77	−3.79	−3.76	−3.70	−3.50	−3.92
* **61** *	108-39-4	3-methylphenol	1.05	−2.11	−2.19	−2.19	−2.20	−2.24	
* **62** *	93-58-3	methyl benzoate	1.11	−2.16	−2.30	−2.29	−2.29	−2.32	
* **63** *	150-68-5	monuron	1.24	−2.63	−2.78	−2.51	−2.60	−2.37	
* **64** *	123-35-3	myrcene	3.01	−0.69	−0.56	−0.77	−0.82	−0.87	
* **65** *	95-53-4	o-toluidine	0.65	−2.53	−2.51	−2.49	−2.63	−2.37	
* **66** *	93-55-0	propiophenone	1.16	−2.10	−2.17	−2.21	−2.32	−2.12	
* **67** *	103-65-1	propylbenzene	2.29	−1.03	−1.16	−1.20	−1.28	−1.24	
* **68** *	106-42-3	p-xylene	1.83	−1.31	−1.46	−1.46	−1.57	−1.47	
* **69** *	289-95-2	pyrimidine	−0.50	−3.52	−3.28	−3.22	−3.31	−3.13	
* **70** *	120-80-9	pyrocatechol	0.49	−2.84	−2.75	−2.76	−2.63	−2.90	
* **71** *	109-97-7	pyrrole	0.18	−2.68	−2.64	−2.59	−2.65	−2.60	
* **72** *	91-22-5	quinoline	2.07	−2.18	−1.68	−1.51	−1.53	−1.59	
* **73** *	108-46-3	resorcinol	0.40	−2.89	−2.83	−2.82	−2.71	−2.95	−3.62
* **74** *	62-56-6	thiourea	−0.77	−3.95	−3.87	−3.96	−3.45	−4.43	
* **75** *	89-83-8	thymol	2.19	−1.45	−1.52	−1.57	−1.55	−1.66	−1.28
* **76** *	1009-14-9	valerophenone	2.01	−1.62	−1.63	−1.73	−1.80	−1.70	
* **77** *	109-66-0	pentane	2.28	−0.96	−0.89	−0.95	−0.87	−1.24	
* **78** *	75-09-2	dichloromethane	0.22	−2.45	−2.30	−2.34	−2.49	−2.28	
* **79** *	67-66-3	chloroform	0.62	−2.17	−2.12	−2.12	−2.26	−2.08	
* **80** *	56-23-5	carbon tetrachloride	1.61	−1.79	−1.48	−1.46	−1.50	−1.58	
* **81** *	1300-21-6	1,2-dichloroethane	0.34	−2.38	−2.28	−2.33	−2.51	−2.22	
* **82** *	79-34-5	1,1,2,2-tetrachloroethane	1.28	−2.16	−1.77	−1.78	−1.93	−1.75	
* **83** *	109-69-3	1-chlorobutane	1.05	−1.57	−1.76	−1.87	−1.99	−1.86	
* **84** *	60-29-7	diethyl ether	−0.06	−2.63	−2.69	−2.74	−2.91	−2.60	−2.80
* **85** *	111-43-3	dipropyl ether	0.78	−2.03	−2.16	−2.26	−2.41	−2.17	
* **86** *	79-20-9	methyl acetate	−0.66	−3.10	−3.23	−3.27	−3.33	−3.22	
* **87** *	141-78-6	ethyl acetate	−0.25	−2.82	−2.98	−3.04	−3.08	−3.01	
* **88** *	123-86-4	butyl acetate	0.62	−2.27	−2.24	−2.55	−2.55	−2.57	
* **89** *	75-05-8	acetonitrile	−0.70	−3.26	−3.13	−3.17	−3.19	−3.19	
* **90** *	107-12-0	propionitrile	−0.35	−3.01	−2.96	−2.98	−3.00	−3.01	
* **91** *	64-17-5	ethanol	−0.62	−3.27	−3.09	−3.10	−3.16	−3.08	−3.10
* **92** *	71-23-8	1-propanol	−0.42	−2.97	−2.99	−3.03	−3.11	−2.98	−2.92
* **93** *	75-65-0	2-methyl-2-propanol	0.37	−2.99	−2.51	−2.52	−2.52	−2.58	
* **94** *	71-41-0	1-pentanol	0.33	−2.30	−2.53	−2.62	−2.68	−2.60	−2.22
* **95** *	75-85-4	2-methyl-2-butanol	−0.08	−2.51	−2.88	−2.93	−3.05	−2.81	
* **96** *	111-27-3	1-hexanol	0.83	−2.03	−2.21	−2.33	−2.36	−2.35	−1.89
* **97** *	64-18-6	formic acid	−0.65	−3.42	−3.18	−3.21	−3.06	−3.42	
* **98** *	64-19-7	acetic acid	−0.68	−3.25	−3.29	−3.31	−3.21	−3.43	−3.21
* **99** *	79-09-4	propionic acid	−0.08	−3.00	−2.89	−2.94	−2.80	−3.13	
* **100** *	107-92-6	butanoic acid	0.38	−2.77	−2.59	−2.67	−2.51	−2.89	
* **101** *	109-99-9	tetrahydrofuran	−0.18	−2.90	−2.89	−2.79	−2.96	−2.66	
* **102** *	93-89-0	ethyl benzoate	1.48	−1.89	−2.08	−2.09	−2.08	−2.13	
* **103** *	88-73-3	1-chloro-2-nitrobenzene	1.44	−2.20	−2.20	−2.22	−1.98	−2.52	
* **104** *	140-29-4	phenylacetonitrile	0.82	−2.43	−2.45	−2.44	−2.48	−2.42	
* **105** *	91-59-8	2-naphthylamine	1.77	−2.09	−2.08	−1.96	−1.97	−1.98	
* **106** *	108-43-0	3-chlorophenol	1.70	−1.87	−1.73	−1.71	−1.61	−1.91	
* **107** *	99-04-7	3-methylbenzoic acid	1.43	−2.00	−2.17	−2.15	−1.99	−2.36	
* **108** *	100-02-7	4-nitrophenol	1.28	−2.32	−2.52	−2.49	−2.04	−2.97	−2.25
* **109** *	17849-38-6	4-chlorobenzyl alcohol	1.06	−2.30	−2.29	−2.26	−2.32	−2.23	
* **110** *	541-73-1	1,3-dichlorobenzene	2.48	−1.28	−1.04	−0.98	−0.99	−1.14	
* **111** *	108-67-8	1,3,5-trimethylbenzene	2.61	−1.21	−0.98	−0.96	−1.00	−1.07	
* **112** *	142-82-5	heptane	3.20	−0.27	−0.30	−0.41	−0.29	−0.78	
* **113** *	110-54-3	hexane	1.97	−0.70	−1.17	−1.26	−1.28	−1.40	
* **114** *	103-90-2	acetaminophen	0.38	−3.35	−3.24	−3.13	−3.07	−3.12	
* **115** *	66085-59-4	nimodipine	3.06	−3.13	−2.88	−2.90	−2.45	−3.07	
* **116** *	57-83-0	progesterone	2.98	−2.00	−1.88	−1.85	−2.05	−1.52	
* **117** *	19387-91-8	tinidazole	0.26	−4.42	−4.15	−3.99	−3.63	−4.14	
* **118** *	106-49-0	p-toluidine	1.15	−2.48	−2.18	−2.21	−2.17	−2.30	
* **119** *	90-41-5	2-aminobiphenyl	2.13	−1.87	−1.82	−1.81	−1.83	−1.80	
* **120** *	103-69-5	N-ethylaniline	1.06	−2.05	−2.29	−2.23	−2.40	−2.08	
* **121** *	86-55-5	1-naphthoic acid	2.13	−1.71	−1.99	−1.85	−1.72	−2.01	
* **122** *	103-82-2	phenylacetic acid	0.76	−2.63	−2.68	−2.64	−2.58	−2.70	
* **123** *	1878-65-5	3-chlorophenylacetic acid	1.47	−2.37	−2.24	−2.20	−2.07	−2.34	
* **124** *	1821-12-1	4-phenylbutanoic acid	1.52	−2.12	−2.20	−2.23	−2.15	−2.32	
* **125** *	103-29-7	1,2-diphenylethane	3.77	−0.64	−0.53	−0.42	−0.49	−0.49	
* **126** *	58-22-0	testosterone	2.23	−2.22	−2.38	−2.31	−2.51	−1.96	
* **127** *	78-93-3	2-butanone	−0.38	−3.02	−2.91	−3.02	−3.16	−2.90	−2.95
* **128** *	110-86-1	pyridine	0.32	−2.82	−2.56	−2.52	−2.62	−2.47	
* **129** *	123-07-9	4-ethylphenol	1.54	−1.78	−1.88	−1.90	−1.89	−1.99	−1.46
* **130** *	645-56-7	4-propylphenol	2.02	−1.44	−1.57	−1.62	−1.58	−1.75	
* **131** *	1638-22-8	4-butylphenol	2.51	−1.22	−1.24	−1.33	−1.26	−1.50	
* **132** *	371-41-5	4-fluorophenol	0.96	−2.26	−2.14	−2.18	−2.15	−2.29	
* **133** *	106-41-2	4-bromophenol	1.81	−2.05	−1.74	−1.67	−1.59	−1.85	−1.44
* **134** *	540-38-5	4-iodophenol	2.10	−2.11	−1.65	−1.54	−1.47	−1.71	
* **135** *	103-79-7	benzyl methyl ketone	0.38	−2.60	−2.76	−2.78	−3.01	−2.52	
* **136** *	72509-76-3	felodipine	3.47	−2.40	−1.91	−1.86	−1.74	−1.84	
* **137** *	63675-72-9	nisoldipine	3.26	−2.82	−2.53	−2.56	−2.09	−2.80	
* **138** *	103890-78-4	lacidipine	4.00	−1.91	−2.04	−2.23	−2.11	−2.06	
* **139** *	738-70-5	trimethoprim	0.95	−3.83	−4.05	−3.88	−3.59	−3.87	
* **140** *	50-24-8	prednisolone	1.65	−3.75	−3.54	−3.50	−3.38	−3.32	
* **141** *	50-78-2	acetylsalicylic acid	0.82	−3.03	−3.01	−2.98	−2.73	−3.16	
* **142** *	65-85-0	benzoic acid	1.05	−2.25	−2.38	−2.36	−2.21	−2.56	
* **143** *	140-65-8	pramocaine	2.56	−2.17	−2.04	−2.02	−2.25	−1.67	
* **144** *	67-64-1	acetone	−0.75	−3.29	−3.13	−3.22	−3.37	−3.09	−3.21
* **145** *	123-30-8	4-aminophenol	−0.20	−3.39	−3.33	−3.36	−3.32	−3.35	
* **146** *	93-60-7	methyl nicotinate	0.27	−3.02	−3.04	−3.00	−2.99	−2.98	
* **147** *	68-12-2	N,N-dimethylformamide	−0.47	−3.89	−3.11	−3.11	−3.22	−3.01	
* **148** *	26839-75-8	timolol	0.90	−3.36	−3.82	−3.97	−3.69	−3.94	
* **149** *	2180-92-9	bupivacaine	1.88	−2.16	−2.56	−2.61	−2.98	−2.04	
* **150** *	721-50-6	prilocaine	0.99	−2.64	−3.01	−3.01	−3.20	−2.66	
* **151** *	29122-68-7	atenolol	0.65	−4.19	−3.71	−3.80	−3.70	−3.64	
* **152** *	525-66-6	propranolol	2.20	−1.95	−1.90	−2.30	−2.43	−2.05	
* **153** *	321-97-1	pseudoephedrine	0.36	−2.98	−3.07	−3.11	−3.30	−2.81	
* **154** *	37517-30-9	acebutolol	1.57	−3.56	−3.42	−3.48	−3.43	−3.23	
* **155** *	13655-52-2	alprenolol	2.08	−2.14	−2.16	−2.33	−2.43	−2.11	
* **156** *	37350-58-6	metoprolol	1.21	−3.06	−3.00	−3.10	−3.27	−2.72	
* **157** *	6452-71-7	oxprenolol	1.55	−2.90	−2.62	−2.84	−2.96	−2.55	
* **158** *	18559-94-9	albuterol	0.48	−3.73	−3.61	−3.70	−3.68	−3.50	
* **159** *	54910-89-3	fluoxetine	2.98	−2.00	−1.50	−1.54	−1.74	−1.28	
* **160** *	52-53-9	verapamil	2.76	−2.84	−2.94	−2.98	−3.38	−2.19	

Where: log ***k_IAM_***—IAM HPLC retention factors [46]; log ***K_p_***^exp^—experimental values [29]; log ***K_p_***^EPI^—values calculated using DERMWIN software [12]; and log ***K_p_***^(9)^ and log ***K_p_***^(11)^ to log ***K_p_***^(13)^—values calculated according to Equations (9) and (11)–(13).

**Table 2:** Calculated descriptors for compounds ***1*** to ***160***.

		*PSA*	*FRB*	*HA*	*V_M_*	*α*	*E_HOMO_*	*E_LUMO_*	*S_a_*
* **1** *	Acetanilide	29.1	1	2	122.5	16.07	−8.82	−0.03	312.2
* **2** *	Acetophenone	17.1	1	1	121.0	14.38	−10.00	−0.44	293.2
* **3** *	2-aminophenol	46.3	2	2	90.1	12.83	−8.10	0.52	264.6
* **4** *	Anisole	9.2	1	1	113.4	13.05	−9.11	0.35	283.9
* **5** *	Benzaldehyde	17.1	1	1	101.1	13.08	−10.05	−0.48	266.7
* **6** *	Benzamide	43.1	1	2	108.1	13.95	−9.72	−0.36	286.2
* **7** *	Benzene	0.0	0	0	89.4	10.41	−9.75	0.40	241.1
* **8** *	Benzonitrile	23.8	0	1	100.0	12.42	−10.10	−0.58	271.7
* **9** *	Benzyl alcohol	20.2	2	1	109.3	12.09	−9.58	0.33	281.5
* **10** *	Biphenyl	0.0	0	0	154.7	20.16	−8.92	−0.36	345.0
* **11** *	1-bromonaphtalene	0.0	0	0	139.7	20.53	−8.99	−0.65	328.6
* **12** *	3-bromophenol	20.2	1	1	104.1	14.20	−9.41	−0.13	282.5
* **13** *	Cinnamyl alcohol	20.2	3	1	127.9	17.32	−8.95	−0.15	329.7
* **14** *	Coumarin	26.3	0	2	117.1	15.76	−9.45	−0.99	305.5
* **15** *	Cyclohexanone	17.1	0	1	103.0	11.02	−10.48	0.85	256.1
* **16** *	Diethyl phtalate	52.6	6	4	198.2	23.42	−10.24	−0.93	407.7
* **17** *	2,6-dimethylphenol	20.2	1	1	120.4	14.98	−8.96	0.29	296.7
* **18** *	4-hydroxybenzyl alcohol	40.5	3	2	101.7	13.71	−9.06	0.31	291.5
* **19** *	2-methylphenol	20.2	1	1	104.1	13.07	−9.06	0.28	275.6
* **20** *	4-methylphenol	20.2	1	1	104.1	13.07	−8.95	0.33	274.0
* **21** *	Naphtalene	0.0	0	0	123.6	17.48	−8.84	−0.41	299.8
* **22** *	1-naphthol	20.2	1	1	122.0	18.23	−8.54	−0.36	308.8
* **23** *	2-naphthol	20.2	1	1	122.0	18.23	−8.72	−0.45	311.6
* **24** *	2-nitroaniline	71.8	2	4	103.6	14.68	−8.75	−0.82	306.6
* **25** *	4-nitroaniline	71.8	2	4	103.6	14.68	−9.00	−0.78	291.6
* **26** *	Nitrobenzene	45.8	1	3	101.3	13.00	−10.60	−1.14	273.3
* **27** *	4-nitrobenzyl alcohol	66.1	3	4	115.1	15.56	−10.52	−1.13	315.5
* **28** *	1-nitrobutane	45.8	3	3	107.3	10.55	−12.01	0.06	275.0
* **29** *	4-nitrotoluene	45.8	1	3	117.6	14.91	−10.47	−1.11	299.3
* **30** *	Phenol	20.2	1	1	87.9	11.15	−9.18	0.29	248.4
* **31** *	2-phenylethanol	20.2	3	1	119.8	14.80	−9.55	0.29	312.0
* **32** *	4-phenylphenol	20.2	2	1	153.2	20.90	−8.64	−0.34	355.8
* **33** *	Toluene	0.0	0	0	105.7	12.32	−9.44	0.45	265.6
* **34** *	4-chlorophenol	20.2	1	1	99.8	13.09	−9.01	0.05	272.4
* **35** *	2,3-benzofuran	13.1	0	1	106.3	14.43	−9.07	−0.15	216.7
* **36** *	2,3-dimethylphenol	20.2	1	1	120.4	14.98	−9.00	0.29	293.8
* **37** *	2,4-dimethylphenol	20.2	1	1	120.4	14.98	−8.86	0.31	301.2
* **38** *	2-nitroanisole	55.1	2	4	125.3	15.65	−10.29	−0.89	316.0
* **39** *	2-chloroaniline	26.0	1	1	103.7	14.03	−8.17	0.31	277.6
* **40** *	3-nitroaniline	71.8	2	4	103.6	14.68	−9.28	−1.06	291.8
* **41** *	4-chloroacetanilide	29.1	1	2	134.5	18.01	−8.67	−0.06	341.5
* **42** *	4-chloroaniline	26.0	1	1	103.7	14.03	−8.11	0.38	282.0
* **43** *	Aniline	26.0	1	1	91.7	12.09	−8.07	0.62	257.9
* **44** *	Antipyrine	23.6	1	3	162.8	21.63	−9.04	−0.33	383.0
* **45** *	Benzophenone	17.1	2	1	167.6	22.22	−9.94	−0.67	374.7
* **46** *	Benzyl benzoate	26.3	4	2	188.0	24.78	−9.61	−0.34	438.6
* **47** *	Bromobenzene	0.0	0	0	105.6	13.46	−9.81	−0.05	273.0
* **48** *	Butylbenzene	0.0	3	0	155.3	17.87	−9.51	0.36	357.3
* **49** *	Butyrophenone	17.1	3	1	154.0	18.06	−9.99	−0.09	363.0
* **50** *	Caffeine	53.5	0	6	133.4	19.97	−8.89	−0.49	364.0
* **51** *	Chlorobenzene	0.0	0	0	101.4	12.36	−9.39	0.06	264.1
* **52** *	Corticosterone	74.6	4	4	284.3	37.27	−10.21	−0.15	531.3
* **53** *	Cortisone	91.7	4	5	280.3	37.33	−10.09	−0.07	533.8
* **54** *	Estradiol	40.5	2	2	232.6	31.52	−8.86	−0.35	468.3
* **55** *	Estriol	60.7	3	3	229.6	32.15	−8.88	0.34	481.2
* **56** *	Ethylbenzene	0.0	1	0	122.3	14.19	−9.51	0.38	294.0
* **57** *	Furan	13.1	0	1	72.2	7.35	−9.38	0.61	208.2
* **58** *	Geraniol	20.2	5	1	177.9	19.71	−9.33	0.99	397.4
* **59** *	Heptanophenone	17.1	6	1	203.5	23.57	−10.00	−0.42	438.5
* **60** *	Hydrocortisone	94.8	5	5	281.4	37.89	−10.25	−0.18	542.9
* **61** *	3-methylphenol	20.2	1	1	104.1	13.07	−9.09	0.29	275.6
* **62** *	Methyl benzoate	26.3	2	2	127.3	15.07	−10.11	−0.46	314.4
* **63** *	Monuron	32.2	1	3	158.2	21.32	−9.13	−0.20	387.9
* **64** *	Myrcene	0.0	4	0	177.0	18.86	−9.28	0.29	374.8
* **65** *	o-toluidine	16.0	1	1	108.0	14.00	−7.99	0.59	276.9
* **66** *	Propiophenone	17.1	2	1	137.5	16.22	−9.99	−0.42	322.0
* **67** *	Propylbenzene	0.0	2	0	138.8	16.03	−9.51	0.37	326.6
* **68** *	p-xylene	0.0	0	0	122.0	14.23	−9.18	0.36	289.8
* **69** *	Pyrimidine	25.8	0	2	75.9	8.89	−10.29	−0.41	226.2
* **70** *	Pyrocatechol	40.5	2	2	86.3	11.90	−9.07	0.26	257.3
* **71** *	Pyrrole	15.8	0	1	67.8	8.20	−8.93	1.11	216.7
* **72** *	Quinoline	12.9	0	1	116.8	16.72	−9.24	−0.65	297.7
* **73** *	Resorcinol	40.5	2	2	86.3	11.90	−9.06	0.27	259.6
* **74** *	Thiourea	88.7	0	2	29.9	7.26	−8.62	−0.27	212.2
* **75** *	Thymol	20.2	2	1	154.2	18.69	−9.00	0.28	346.2
* **76** *	Valerophenone	17.1	4	1	170.5	19.89	−9.99	−0.42	379.6
* **77** *	Pentane	0.0	2	0	111.1	10.00	−11.30	3.45	266.9
* **78** *	Dichloromethane	0.0	0	0	67.8	6.49	−10.48	0.52	199.3
* **79** *	Chloroform	0.0	0	0	79.6	8.40	−10.88	−0.12	224.5
* **80** *	Carbon tetrachloride	0.0	0	0	90.6	10.32	−10.99	−0.63	244.2
* **81** *	1,2-dichloroethane	0.0	1	0	84.3	8.33	−10.69	0.54	231.1
* **82** *	1,1,2,2,-tetrachloroethane	0.0	1	0	107.8	12.14	−10.74	0.02	267.0
* **83** *	1-chlorobutane	0.0	2	0	106.0	10.08	−10.31	1.23	264.9
* **84** *	Diethyl ether	9.2	2	1	100.9	8.85	−10.48	2.86	260.9
* **85** *	Dipropyl ether	9.2	4	1	133.9	12.52	−10.49	2.76	317.2
* **86** *	Methyl acetate	26.3	1	2	81.5	7.03	−11.26	1.02	228.3
* **87** *	Ethyl acetate	26.3	2	2	98.0	8.83	−11.24	1.06	263.0
* **88** *	Butyl acetate	26.3	4	2	131.0	12.54	−11.25	1.05	232.4
* **89** *	Acetonitrile	23.8	0	1	54.9	4.45	−12.33	1.40	176.4
* **90** *	Propionitrile	23.8	0	1	71.4	6.29	−12.01	1.42	211.9
* **91** *	Ethanol	20.2	1	1	59.1	5.09	−10.90	3.33	191.0
* **92** *	1-propanol	20.2	2	1	75.6	6.93	−10.88	3.23	220.6
* **93** *	2-methyl-2-propanol	20.2	1	1	92.1	8.75	−11.28	3.26	244.4
* **94** *	1-pentanol	20.2	4	1	108.6	10.60	−10.89	3.11	281.4
* **95** *	2-methyl-2-butanol	20.2	2	1	108.6	10.59	−11.13	3.17	264.1
* **96** *	1-hexanol	20.2	5	1	125.1	12.44	−10.89	3.09	313.0
* **97** *	Formic acid	37.3	0	2	39.9	3.33	−11.57	0.97	158.3
* **98** *	Acetic acid	37.3	0	2	56.2	5.11	−11.43	0.93	192.8
* **99** *	Propionic acid	37.3	1	2	72.7	6.94	−11.31	0.96	222.4
* **100** *	Butanoic acid	37.3	2	2	89.2	8.78	−11.34	0.97	249.1
* **101** *	Tetrahydrofuran	9.2	0	1	79.8	7.95	−10.28	3.29	225.0
* **102** *	Ethyl benzoate	26.3	3	2	143.8	16.91	−10.08	−0.43	348.2
* **103** *	2-chloro-1-nitrobenzene	45.8	3	3	113.2	14.94	−9.94	−1.27	290.8
* **104** *	Phenylacetonitrile	23.8	1	1	115.7	14.16	−9.99	−0.09	300.7
* **105** *	2-naphthylamine	26.0	1	1	125.8	19.16	−7.92	−0.21	319.6
* **106** *	3-chlorophenol	20.2	1	1	99.8	13.09	−9.24	−0.01	272.6
* **107** *	3-methylbenzoic acid	37.3	1	2	118.2	15.07	−9.82	−0.49	304.5
* **108** *	4-nitrophenol	66.1	2	4	99.7	13.75	−10.17	−1.08	283.4
* **109** *	4-chlorobenzyl alcohol	20.2	2	1	115.2	14.91	−9.28	0.02	306.2
* **110** *	1,3-dichlorobenzene	0.0	0	0	113.3	14.29	−9.42	−0.19	288.6
* **111** *	1,3,5-trimethylbenzene	0.0	0	0	138.3	16.15	−9.28	0.44	320.3
* **112** *	Heptane	0.0	4	0	144.1	13.67	−11.27	3.30	327.7
* **113** *	Hexane	0.0	3	0	127.6	11.83	−11.28	3.36	297.7
* **114** *	Acetaminophen	49.3	1	3	121.0	16.81	−8.56	−0.02	323.6
* **115** *	Nimodipine	119.7	10	9	345.0	42.87	−9.21	−0.66	682.0
* **116** *	Progesterone	34.1	1	2	289.0	36.06	−10.14	−0.10	514.8
* **117** *	Tinidazole	101.2	5	7	172.4	23.36	−10.47	−1.21	432.8
* **118** *	p-toluidine	26.0	1	1	108.0	14.00	−7.95	0.64	285.0
* **119** *	2-aminobiphenyl	26.0	2	1	157.0	21.84	−7.98	−0.21	356.8
* **120** *	N-ethylaniline	12.0	2	1	125.4	16.05	−8.50	0.45	322.8
* **121** *	1-naphthoic acid	37.3	1	2	136.1	20.23	−9.13	−0.97	334.0
* **122** *	Phenylacetic acid	37.3	2	2	116.9	14.81	−9.81	0.22	313.7
* **123** *	3-chlorophenylacetic acid	37.3	2	2	128.8	16.75	−9.52	−0.20	337.0
* **124** *	4-phenylbutanoic acid	37.3	4	2	149.9	18.49	−9.67	0.21	372.0
* **125** *	1,2-diphenylethane	0.0	2	0	183.0	23.90	−9.48	0.32	402.0
* **126** *	Testosterone	37.3	1	2	257.0	32.95	−10.12	−0.09	477.5
* **127** *	2-butanone	17.1	1	1	91.7	8.17	−10.65	0.83	239.1
* **128** *	Pyridine	12.9	0	1	82.7	9.65	−10.10	0.01	231.9
* **129** *	4-ethylphenol	20.2	2	1	120.7	14.94	−9.00	0.32	305.7
* **130** *	4-propylphenol	20.2	3	1	137.2	16.78	−9.00	0.32	335.7
* **131** *	4-butylphenol	20.2	4	1	153.7	18.61	−8.99	0.32	360.8
* **132** *	4-fluorophenol	20.2	1	1	92.1	11.15	−9.27	−0.06	254.4
* **133** *	4-bromophenol	20.2	1	1	104.1	14.20	−9.31	−0.03	283.6
* **134** *	4-iodophenol	20.2	1	1	109.9	16.27	−8.84	−0.41	289.8
* **135** *	Benzyl methyl ketone	17.1	2	1	135.9	16.04	−9.71	0.07	320.4
* **136** *	Felodipine	64.6	6	5	300.8	37.97	−8.87	−0.18	588.5
* **137** *	Nisoldipine	110.5	8	8	322.2	40.34	−9.15	−0.73	628.4
* **138** *	Lacidipine	90.9	11	7	404.0	50.26	−8.91	−1.29	706.6
* **139** *	Trimethoprin	105.5	5	7	231.9	31.82	−8.73	−0.07	519.0
* **140** *	Prednisolone	94.8	5	5	274.7	37.85	−10.07	−0.42	527.4
* **141** *	Acetylsalicylic acid	63.6	3	4	139.6	17.65	−10.19	−0.54	355.9
* **142** *	Benzoic acid	37.3	1	2	102.0	13.15	−10.13	−0.53	278.6
* **143** *	Pramocaine	30.9	9	4	284.6	33.45	−8.78	0.24	595.7
* **144** *	Acetone	17.1	0	1	75.2	6.33	−10.76	0.80	206.7
* **145** *	4-aminophenol	46.3	2	2	90.1	12.83	−7.84	0.46	267.6
* **146** *	Methyl nicotinate	39.2	2	3	120.6	14.32	−10.44	−0.80	310.4
* **147** *	N,N-dimethylformamide	20.3	0	2	82.6	7.87	−9.26	1.36	228.9
* **148** *	Timolol	108.0	8	7	258.5	32.57	−9.24	−1.05	520.8
* **149** *	Bupivacaine	32.3	5	3	279.2	35.13	−7.46	−0.40	514.1
* **150** *	Prilocaine	41.1	5	3	214.0	26.73	−9.23	0.18	449.6
* **151** *	Atenolol	84.6	9	5	236.7	29.44	−9.30	−0.03	541.0
* **152** *	Propranolol	41.5	7	3	237.2	31.31	−8.62	−0.43	307.7
* **153** *	Pseudoefedrine	32.3	4	2	162.7	19.88	−9.44	0.29	371.4
* **154** *	Acebutolol	87.7	11	6	300.7	37.55	−9.15	−0.43	642.5
* **155** *	Alprenolol	41.5	9	3	247.5	29.75	−9.12	0.26	506.5
* **156** *	Metoprolol	50.7	10	4	258.7	30.55	−9.00	−0.27	569.8
* **157** *	Oxprenolol	50.7	10	4	255.2	30.45	−9.21	0.12	496.3
* **158** *	Albuterol	72.7	8	4	207.6	26.86	−8.97	0.16	474.7
* **159** *	Fluoxetine	21.3	6	2	266.7	31.67	−9.44	−0.39	541.6
* **160** *	Verapamil	64.0	13	6	429.4	52.28	−8.78	−0.95	817.4

## Data Availability

Data generated in this study can be found in this manuscript.
